# Supplementary material for: Pan-cancer proteomic map of 949 human cell lines
Source: Cancer Cell. 2022 Aug 8;40(8):835–849.e8. doi: 10.1016/j.ccell.2022.06.010 (PMC9387775; doi:10.1016/j.ccell.2022.06.010)
Supplement: Document S1. Figures S1–S5 [file mmc1.pdf]

Cancer Cell, Volume *40*

Supplemental information

Pan-cancer proteomic

map of 949 human cell lines

Emanuel Gonçalves, Rebecca C. Poulos, Zhaoxiang Cai, Syd Barthorpe, Srikanth S. Manda, Natasha Lucas, Alexandra Beck, Daniel Bucio-Noble, Michael Dausmann, Caitlin Hall, Michael Hecker, Jennifer Koh, Howard Lightfoot, Sadia Mahboob, Iman Mali, James Morris, Laura Richardson, Akila J. Seneviratne, Rebecca Shepherd, Erin Sykes, Frances Thomas, Sara Valentini, Steven G. Williams, Yangxiu Wu, Dylan Xavier, Karen L. MacKenzie, Peter G. Hains, Brett Tully, Phillip J. Robinson, Qing Zhong, Mathew J. Garnett, and Roger R. Reddel

Supplementary Figures

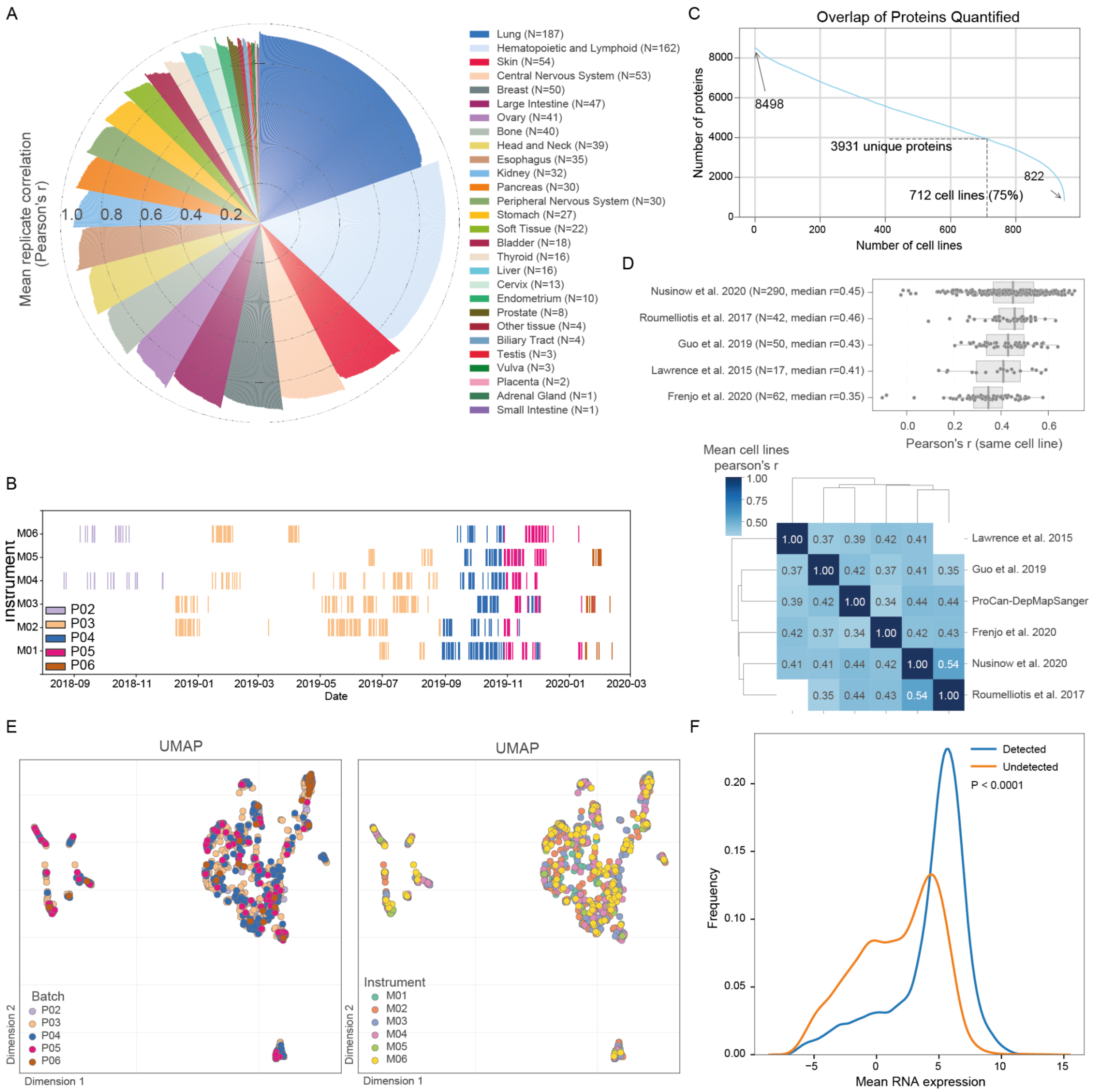

Figure S1. A pan-cancer proteomic map of 949 human cancer cell lines by Data Independent Acquisition Mass Spectrometry (DIA-MS), Related to Figure 1. A, *Mean Pearson's r for replicates of each cancer cell line, colored by tissue of origin.* ***B,*** *Timeline of MS data acquisition across mass spectrometers, colored according to processing batches (P02 - P06).* ***C,*** *Frequency of proteins identified across the 949 cancer cell lines.* ***D,*** *Upper panel, correlation by Pearson's r of ProCan-DepMapSanger dataset against independent proteomic datasets that comprise subsets of the same cell lines. Box-and-whisker plots indicate*

interquartile range (IQR) with a line at the median. Whiskers represent the minimum and maximum values at 1.5 x IQRs. Lower panel, heatmap and dendrogram (average method with euclidean metric) of the mean pairwise correlations between the same cell lines in different studies. ***E,*** *Uniform Manifold Approximation and Projection (UMAP) dimensionality reduction of cell line proteomes colored by processing batches (left) and mass spectrometer (right).* ***F****, Distribution of mean RNA-seq expression for genes corresponding to proteins that were detected (blue) or undetected (orange) in the ProCan-DepMapSanger dataset. Significance is indicated by Mann-Whitney U test.*

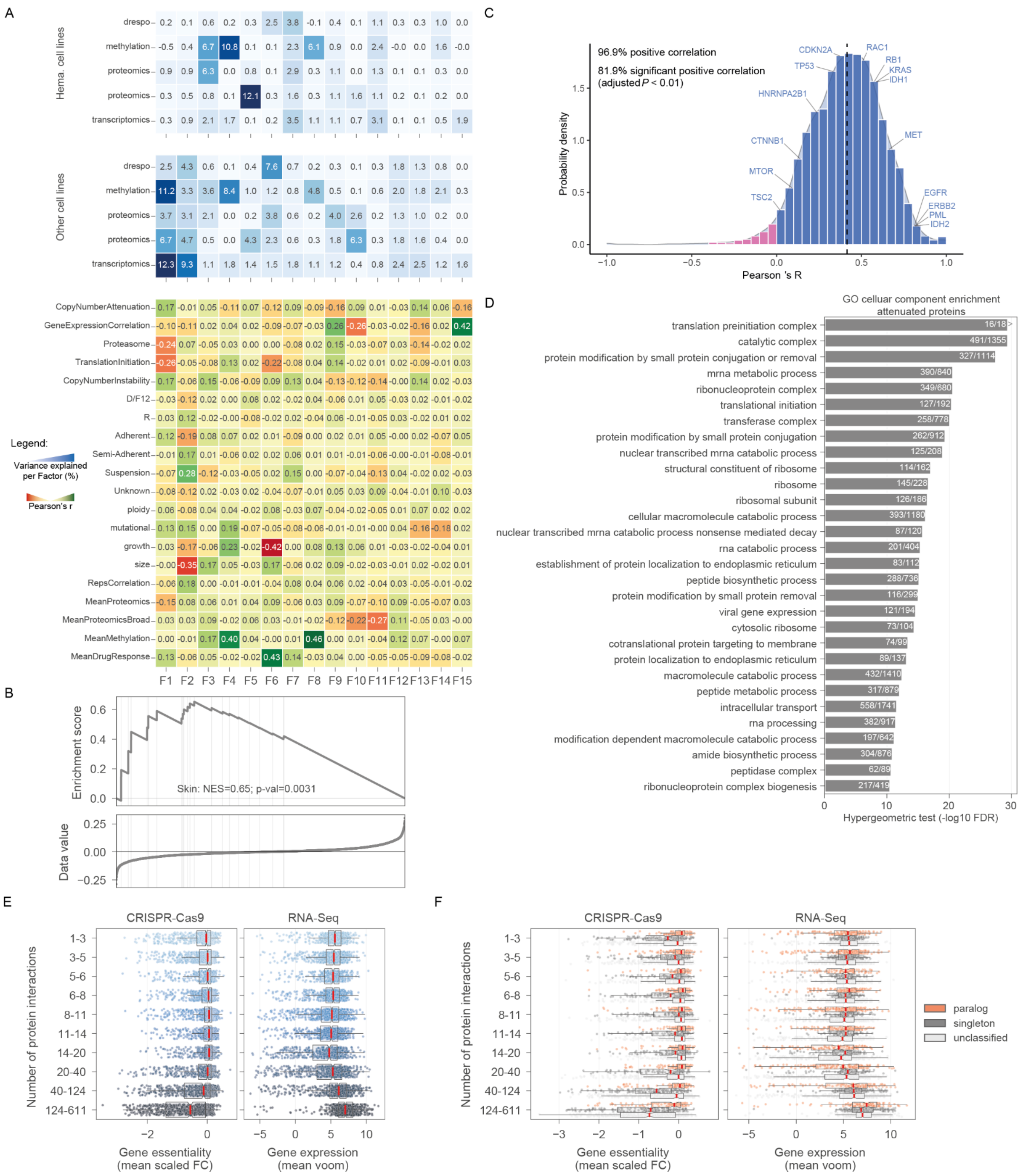

Figure S2. Multi-Omics Factor Analysis (MOFA) and post-transcriptional regulation, Related to Figure 3. A, *Similar to* ***Figure 3A****, MOFA factors across molecular and phenotypic cancer cell line datasets, including ProCan-DepMapSanger. Hematopoietic and lymphoid cells are grouped and trained separately from the other cell lines corresponding to each factor (column). The upper two heatmaps (blue) report the portion of variance explained by each factor (columns) in each dataset. The lower heatmap reports Pearson's r between each learned factor*

and various molecular characteristics of the cancer cell lines. ***B,*** *Gene Set Enrichment Analysis (GSEA) demonstrating enrichment of skin cell type-enriched proteins in MOFA Factor 12.* ***C,*** *Per-gene Pearson's r between protein and RNA expression for all proteins quantified. Mean correlation (r = 0.42) is indicated by a dashed line. The locations of several cancer-related genes are shown.* ***D,*** *Enrichment analysis of proteins that were highly attenuated (n = 1,215), as determined by correlations between protein and copy number, and gene expression and copy number, in terms of Pearson's r. The top most significantly enriched sets are annotated.* ***E, F,*** *Proteins were grouped by their number of significant positive correlations by Pearson's r for putative protein interactions (FDR < 5%, r > 0.5). For each protein, the respective mean scaled CRISPR-Cas9 gene essentiality fold-change (FC) and gene expression (RNA-seq voom) measurement are calculated. Box-and-whisker plots indicate interquartile range (IQR) with a line at the median. Whiskers represent the minimum and maximum values at 1.5 x IQRs.* ***E,*** *The distribution across all proteins is represented and* ***F,*** *proteins are subgrouped into paralog, singleton and unclassified, as previously determined (Dandage and Landry, 2019).*

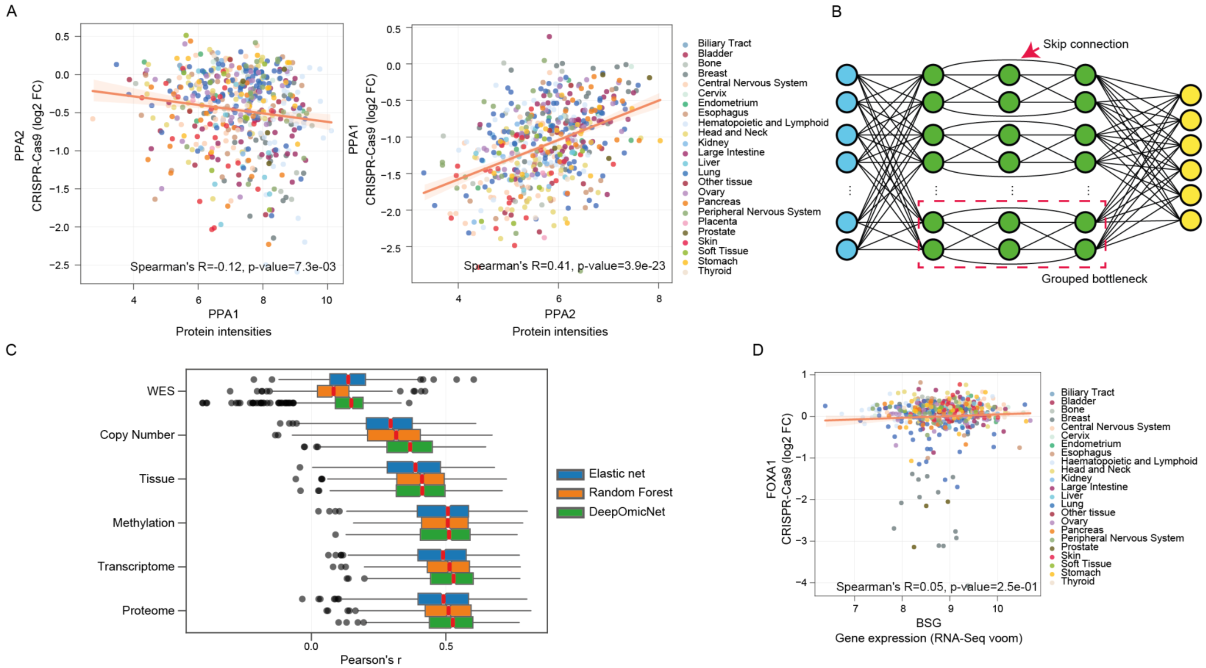

Figure S3. Drug-protein and CRISPR-Cas9-protein associations and Deep Proteomic Marker (DeeProM) analysis pipeline, Related to Figure 4 and Figure 5. A, *Synthetic lethal association between PPA2 and PPA1. Left panel, scatter plot between protein intensities of PPA1 and PPA2 CRISPR-Cas9 gene essentiality scores. Right panel, scatter plot between protein intensities of PPA2 and CRISPR-Cas9 gene essentiality scores of PPA1. Cell lines are colored by tissue types.* ***B,*** *Neural network architecture of DeepOmicNet. In addition to the basic multilayer perceptron (MLP) architecture, skip connections with grouped bottlenecks were added to provide a deeper and wider network. Blue circles represent input neurons, green represents hidden layer neurons and yellow represents output neurons.* ***C,*** *Comparison of observed drug responses and predicted drug responses by DeeProM, elastic net and Random Forest. WES, mutation data from whole exome sequencing; Copy number, copy number profiles; Tissue, categorical variable representing the cell line's tissue of origin; Methylation, promoter region methylation level; Transcriptome, RNA-seq data; Proteome, the ProCan-DepMapSanger dataset. Box-and-whisker plots indicate interquartile range (IQR) with a line at the median. Whiskers represent the minimum and maximum values at 1.5 x IQRs.* ***D,*** *Scatter plot between FOXA1 CRISPR-Cas9 gene essentiality scores and BSG gene expression measurements. Data points are colored according to tissue type.*

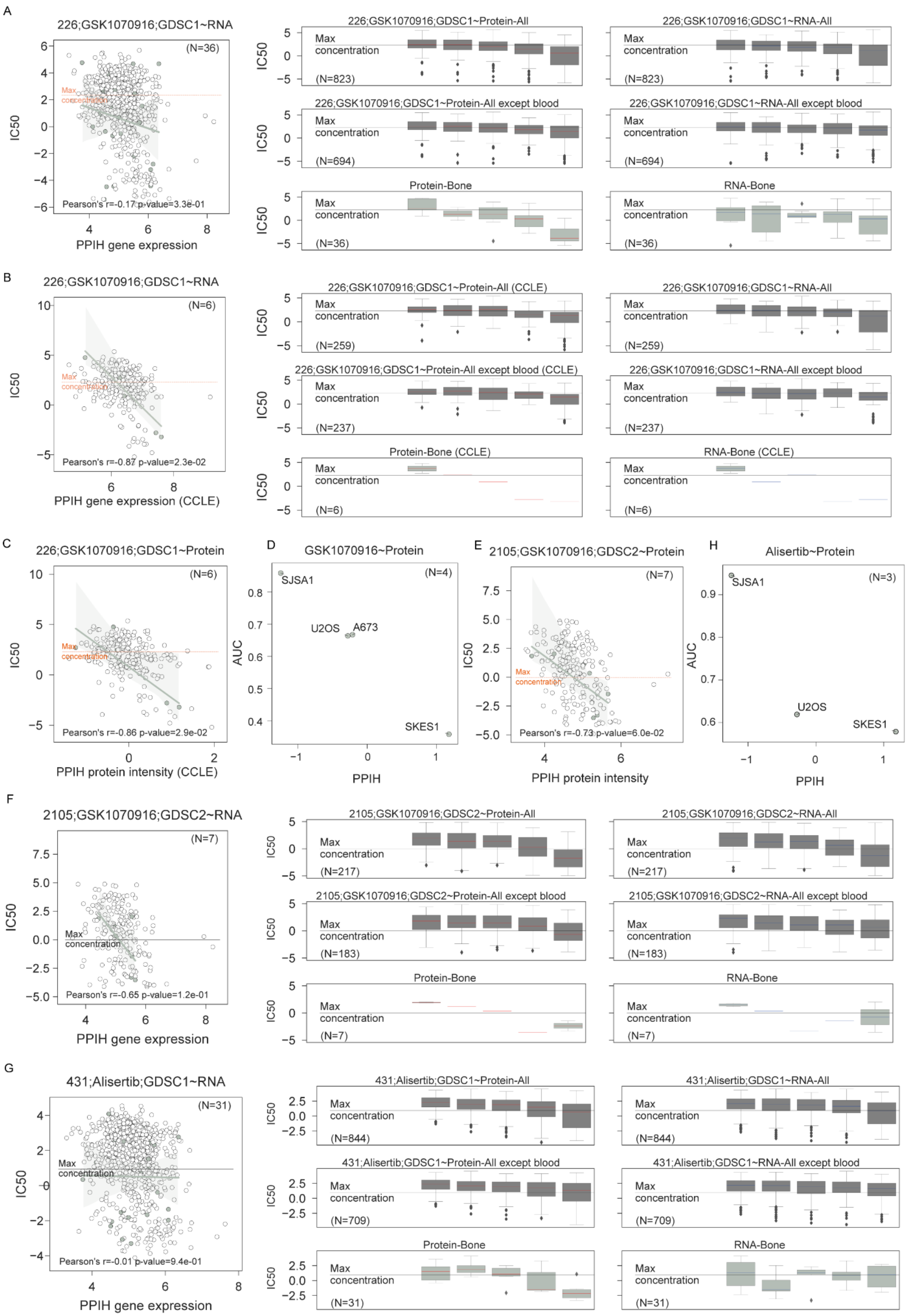
A
226;GSK1070916;GDSC1~RNA
(N=36)
Max concentration
Pearson's r=-0.17 p-value=3.3e-01
IC50
PPIH gene expression
226;GSK1070916;GDSC1~Protein-All
226;GSK1070916;GDSC1~RNA-All
(N=823)
226;GSK1070916;GDSC1~Protein-All except blood
226;GSK1070916;GDSC1~RNA-All except blood
(N=694)
Protein-Bone
RNA-Bone
(N=36)
B
226;GSK1070916;GDSC1~RNA
(N=6)
Pearson's r=-0.87 p-value=2.3e-02
PPIH gene expression (CCLE)
226;GSK1070916;GDSC1~Protein-All (CCLE)
226;GSK1070916;GDSC1~RNA-All
(N=259)
226;GSK1070916;GDSC1~Protein-All except blood (CCLE)
226;GSK1070916;GDSC1~RNA-All except blood
(N=237)
Protein-Bone (CCLE)
RNA-Bone (CCLE)
(N=6)
C
226;GSK1070916;GDSC1~Protein
(N=6)
Pearson's r=-0.86 p-value=2.9e-02
PPIH protein intensity (CCLE)
D
GSK1070916~Protein
(N=4)
SJSA1
U2OS
A673
SKES1
AUC
PPIH
E
2105;GSK1070916;GDSC2~Protein
(N=7)
Pearson's r=-0.73 p-value=6.0e-02
PPIH protein intensity
H
Alisertib~Protein
(N=3)
SJSA1
U2OS
SKES1
AUC
PPIH
F
2105;GSK1070916;GDSC2~RNA
(N=7)
Pearson's r=-0.65 p-value=1.2e-01
PPIH gene expression
2105;GSK1070916;GDSC2~Protein-All
2105;GSK1070916;GDSC2~RNA-All
(N=217)
2105;GSK1070916;GDSC2~Protein-All except blood
2105;GSK1070916;GDSC2~RNA-All except blood
(N=183)
Protein-Bone
RNA-Bone
(N=7)
G
431;Alisertib;GDSC1~RNA
(N=31)
Pearson's r=-0.01 p-value=9.4e-01
PPIH gene expression
431;Alisertib;GDSC1~Protein-All
431;Alisertib;GDSC1~RNA-All
(N=844)
431;Alisertib;GDSC1~Protein-All except blood
431;Alisertib;GDSC1~RNA-All except blood
(N=709)
Protein-Bone
RNA-Bone
(N=31)

Figure S4. Tissue-level protein biomarkers for GSK1070916 across datasets, Related to Figure 5. A-C, E-G *Scatter plots show the relationship between the drug and the protein biomarker, using either protein abundance or underlying RNA expression in cell lines from bone (green; all other cell lines are shown in gray). The number of cell lines and Pearson's r from the highlighted tissue type are annotated at the top right and bottom left corners, respectively. The dashed line represents the maximum concentration used in the drug response screens. In* ***A***, ***B***, ***F*** *and* ***G***, *Box-and-whisker plots summarize the information from the scatter plots of protein (left) and RNA (right). The protein intensity is divided into five equally spaced quantiles from low to high, and the corresponding* IC_{50} *values in the natural logarithmic scale are shown for each quantile. The first row of plots shows the relationship for all cell lines, and non-hematopoietic cell lines are shown in the second row. The relationship for only cell lines from bone is shown in the third row. Box-and-whisker plots indicate interquartile range (IQR) with a line at the median. Whiskers represent the minimum and maximum values at 1.5 x IQRs.* ***A,*** *The association between PPIH gene expression and GSK1070916 drug response in cell lines from bone.* ***B,*** *Similar to* ***A,*** *instead using CCLE gene expression data.* ***C,*** *Similar to* ***A,*** *instead using the CCLE proteomic dataset.* ***D,*** *The association between PPIH protein abundance and response to GSK1070916 using the CCLE proteomic dataset and PRISM drug response data, with the figure obtained from the DepMap Portal.* ***E,*** *Similar to* **Figure 5D**, *instead using the GDSC2 drug response dataset.* ***F,*** *Similar to* ***E,*** *instead using gene expression data.* ***G,*** *The association between PPIH gene expression and Alisertib drug response from GDSC data in cell lines from bone.* ***H,*** *Similar to* ***D***, *instead showing response to Alisertib.*

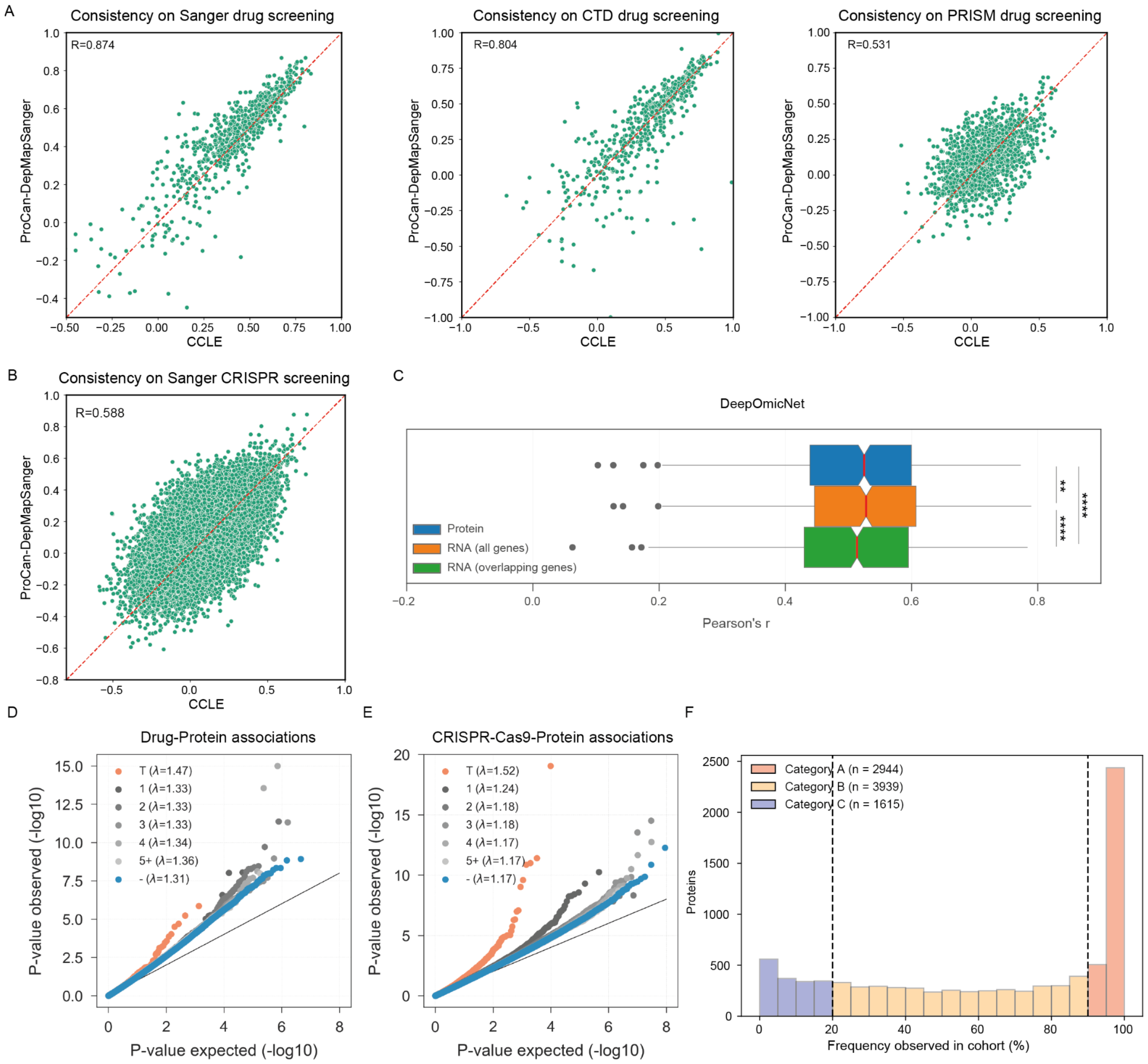

***Figure S5. Predictive power benchmarks and comparisons, Related to Figure 6 and Figure 7. A,** Comparison of DeepOmicNet mean predictive power across three independent drug response datasets, trained using the ProCan-DepMapSanger and the CCLE proteomic dataset**. B,** Similar to **A**, models trained to predict CRISPR-Cas9 gene essentiality profiles. **C,** Comparison of the predictive power of the DeepOmicNet model trained using the ProCan-DepMapSanger dataset and RNA expression data, both for all genes or for only genes that have their corresponding proteins quantified (overlapping genes). **** p-value < 0.0001, ** p-value < 0.01. Significance is by two-tailed paired Student's t-test. Box-and-whisker plots indicate interquartile range (IQR) with a line at the median. Whiskers represent the minimum and maximum values at 1.5 x IQRs. **D,** Drug responses (drug-protein) and **E,** CRISPR-Cas9 gene essentiality (CRISPR-Cas9-protein) associations, identified with linear regression models*

*without taking gene expression as covariates. See **Figure 7C-D** for a general description of the plot. **F,** Histogram showing the distribution of the frequency of proteins detected in each of the cell lines in the cohort, with Category A, B and C proteins indicated. The dashed vertical lines indicate the frequency thresholds for defining the categories.*
